# Kindlin-2 in Sertoli cells is essential for testis development and male fertility in mice

**DOI:** 10.1038/s41419-021-03885-4

**Published:** 2021-06-11

**Authors:** Xiaochun Chi, Weiwei Luo, Jiagui Song, Bing Li, Tiantian Su, Miao Yu, Tianzhuo Wang, Zhenbin Wang, Cheng Liu, Zhen Li, Huiying He, Jun Zhan, Hongquan Zhang

**Affiliations:** 1 Department of Human Anatomy, Histology and Embryology, Key Laboratory of Carcinogenesis and Translational Research (Ministry of Education) and State Key Laboratory of Natural and Biomimetic Drugs, Peking University Health Science Center, Beijing, 100191 China; 2 Department of Histology and Embryology, the Fourth Military Medical University, Xi’an, 710032 China; 3 Department of Pathology, School of Basic Medical Sciences, Peking University Health Science Center, Beijing, 100191 China

**Keywords:** Apoptosis, Male factor infertility

## Abstract

Kindlin-2 is known to play important roles in the development of mesoderm-derived tissues including myocardium, smooth muscle, cartilage and blood vessels. However, nothing is known for the role of Kindlin-2 in mesoderm-derived reproductive organs. Here, we report that loss of Kindlin-2 in Sertoli cells caused severe testis hypoplasia, abnormal germ cell development and complete infertility in male mice. Functionally, loss of Kindlin-2 inhibits proliferation, increases apoptosis, impairs phagocytosis in Sertoli cells and destroyed the integration of blood-testis barrier structure in testes. Mechanistically, Kindlin-2 interacts with LATS1 and YAP, the key components of Hippo pathway. Kindlin-2 impedes LATS1 interaction with YAP, and depletion of Kindlin-2 enhances LATS1 interaction with YAP, increases YAP phosphorylation and decreases its nuclear translocation. For clinical relevance, lower Kindlin-2 expression and decreased nucleus localization of YAP was found in SCOS patients. Collectively, we demonstrated that Kindlin-2 in Sertoli cells is essential for sperm development and male reproduction.

## Introduction

Kindlin-2 is an important integrin-binding protein that has been reported to express in mesodermal derived tissues^1^. Global inactivation of Kindlin-2 in mice results in embryonic lethality at embryonic day 7.5 (E7.5), indicating that it plays a critical role in early embryonic development^2^. Conditional ablation of Kindlin-2 in mouse limb and head mesenchymal progenitor cells results in neonatal lethality, severe chondrodysplasia, and complete loss of the skull vault^3^. Postnatal loss of Kindlin-2 from cardiac myocytes leads to progressive heart failure, indicating its importance for normal heart homeostasis^4^. In addition, Loss of Kindlin-2 impairs smooth muscle formation during embryonic development by inducing apoptosis and jeopardizes the contraction of adult smooth muscle cells by affecting the Ca^2+^ influx^5^. What does the role of Kindlin-2 in mesoderm derived reproductive organs? Our previous results suggested that genitourinary tract-specific knockout of Kindlin-2 (cdh16-Cre mediated deletion) may partially affect the reproductive capacity of male mice, which prompted us to study the effects of Kindlin-2 on male reproductive capability.

Infertility is a worldwide problem in both men and women. A severe form of human male infertility is characterized histologically by the apparent lack of all spermatogenic cells in almost all seminiferous tubules, a condition called Sertoli cell-only syndrome (SCOS)^6^. It is estimated that 5–10% of infertile men may have SCOS, in which the only symptom is infertility^7^.

As supporters and nurse cells, Sertoli cells (SCs) play critical roles in testicular development and spermatogenesis^8^. In fetal life, SCs are involved in primordial germ cell (GC) migration and initiate the process of genetic sex determination in the embryo^9^. Within the seminiferous tubules of the adult testis, mature SCs develop complex interactions with each other as well as with adjacent GCs, providing structural support for spermatogenesis^10^. The junctional complexes between SCs, forming the blood-testis barrier (BTB), physically separate the seminiferous tubules into basal and apical compartments^11^. The BTB is a highly dynamic ultrastructure that accommodates the transport of preleptotene spermatocytes during spermatogenesis. Moreover, the BTB protects developing GCs from autoimmune reactions and exogenous toxins^12^. In addition, once GCs enter the apical compartment, they rely solely on the nutritional supply from SCs^13^. Moreover, SCs produce a testicular androgen-binding protein (ABP), also referred to as sex hormone-binding globulin (SHBG), which is necessary for spermatogenesis^14^. Testicular ABP binds testosterone, maintains its high levels inside the tubular compartment, and transports it to the epididymis^15^. In general, impairments in SC structure and function lead to male infertility.

Here, we generated SC-conditional knockout (cKO) Kindlin-2 mice, exhibiting severe testis hypoplasia and complete male mice infertility. Depletion of Kindlin-2 resulted in yes-associated protein (YAP) phosphorylation and retention in the cytoplasm, ultimately leading to reduced SC proliferation and increased apoptosis. Mechanistically, Kindlin-2, functioning as an “insulator”, isolates the interaction between large tumor suppressor kinase 1 (LATS1) and YAP, thus inhibiting YAP phosphorylation by LATS1. In addition, loss of Kindlin-2 is correlated with SCOS in male infertility patients. Our results demonstrated that Kindlin-2 in SCs is indispensable for male reproduction.

## Results

### Conditional knockout of Kindlin-2 in Sertoli cells inhibits postnatal testicular development

To elucidate the role of Kindlin-2 in testicular development and regulation of male fertility, we generated Sertoli cell-specific Kindlin-2 gene knockout (SC-cKO) mice using the Cre-LoxP system (Supplementary Fig. 1A). The genotype was characterized by polymerase chain reaction (PCR) (Supplementary Fig. 1B).

To confirm the specificity and efficiency of Amh promoter-Cre expression, we used Cre reporter *Rosa26*^*mTmG*^ mice to generate *Amh-Cre*; *ROSA26*^*mTmG*^ mice (Supplementary Fig. 1C)^16^. Fluorescence microscopy showed that tdTomato was expressed in the whole testes (i.e., all testicular cells of the testes) of *ROSA26*^*mTmG*^ mice, while eGFP was expressed specifically in Amh promoter-containing cells of *Amh-cre*; *ROSA26*^*mTmG*^ testis with the stromal cells of *Amh-cre* negative cells remaining red, indicating the specificity of *Amh-cre* knockout (Supplementary Fig. 1D).

From general observation, there were no significant differences between the *Amh-Cre*; *Kindlin-2*^*f/f*^ (KO) mice and mice with control genotypes at 8 weeks (8 W), i.e., *Kindlin-2*^*f/f*^, *Amh-Cre*; *Kindlin-2*^*f/+*^, and *Amh-Cre*; *Kindlin-2*^*+/+*^ mice (Supplementary Fig. 1E). Therefore, we used *Kindlin-2*^*f/f*^ (WT) mice as controls in the subsequent experiments. However, KO mice showed much smaller testicles than control mice (8 W) (Supplementary Fig. 1F). The male KO mice also showed smaller epididymis compared to control mice (8 W) (Supplementary Fig. 1G).

**Fig. 1:**
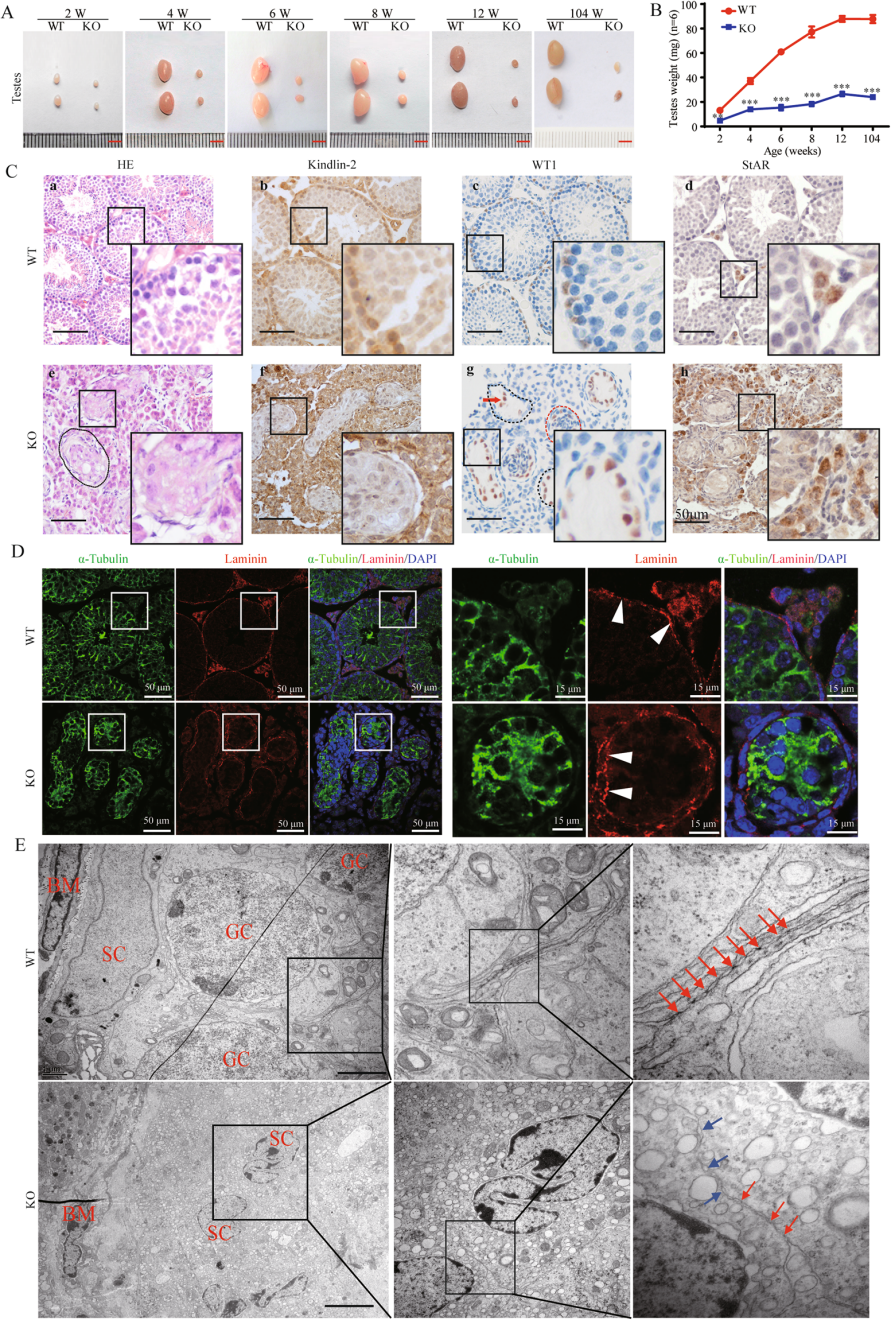
Sertoli cell-specific knockout of Kindlin-2 in mice induced destruction of seminiferous tubules and testicular dysplasia. **A** Gross morphology of testes from WT (refers to *Kindlin-2*^*f/f*^) and KO (refers to *Amh-Cre*; *Kindlin-2*^*f/f*^) mice at 2–12 W. Compared to the WT mice, the size of KO testes is dramatically decreased. Scale bar, 5 mm. **B** Quantification of bilateral testes for mice (mean ± S.D. and *n* = 6). The variance was similar between the groups that are being statistically compared. Statistical analyses were performed using Student’s t-test at indicated time point. The variance was similar between the groups. *p < 0.05, **p < 0.01, ***p < 0.001. **C** Hematoxylin and eosin-staining (HE) and immunohistochemistry (IHC) of Kindlin-2, WT1 (Sertoli cell nucleus marker) and StAR (Leydig cell marker) in 4 W testes. Scale bar, 50 μm. Red arrows indicate the Sertoli cells, red and blank broken lines indicate the testicular tubular lumina. **D** Immunofluorescence of α-Tubulin (green) and laminin (red) in 4 W testes of WT and KO mice. The basement membrane of KO mice was thickener than that of WT mice (white arrow heads) Scale bar, 50 μm. **E** Transmission electron microscopy (TEM) of 8 W testes. SC means Sertoli cell, GC means germinal cell, BM means basement membrane. Scale bar, 2 μm. Red arrows: tight junction in WT testes and a large gap with only a loose cell junction between adjacent SCs in KO testes. Blue arrows: vacuoles located between adjacent SCs in KO testis.

After birth, the testicular volume of KO mice was found to remain low at indicated time, and the testes of KO mice did not develop during 2–6 W (Fig. 1A, B). Testicular underdevelopment was observed in all KO mice (100%) but none (0%) of the mice in the control group. Histological examination revealed that with specific Kindlin-2 KO in SCs, the seminiferous tubules collapsed at 4 W (blank broken lines) (Fig. 1C). However, the sizes of the testicles and epididymis of 2-day-old KO mice were not significantly different from controls (Supplementary Fig. 2A). In addition, the testicular cords of 2-day-old KO mice appeared grossly normal compared with the control group (Supplementary Fig. 2A).

To investigate the changes in the dysgenic testis, we performed immunohistochemical (IHC) and immunofluorescence (IF) analysis of the seminiferous tubules for markers of SCs (WT1 and Tubulin), Leydig cells (StAR), and basement membrane (Laminin). The lumen of the seminiferous tubules was atrophied (Fig. 1C) and the basement membrane (Laminin) was thickened in KO mice (Fig. 1D). However, the proportion of Leydig cells was increased in the KO testis compared to WT controls (Fig. 1C—d and h).

In addition, the ultrastructure of SCs from 4 W-old mice was examined by electron microscopy. Compared to WT mice, KO mice showed a disordered distribution of SCs in the seminiferous tubules and no GCs were found. Moreover, the SCs of KO mice showed an irregularly shaped and pyknotic nucleus. Further, KO mice showed large gaps and loose cell junctions between adjacent SCs (Fig. 1E).

### Kindlin-2 knockout in Sertoli cells leads to infertility in male mice

**Fig. 2:**
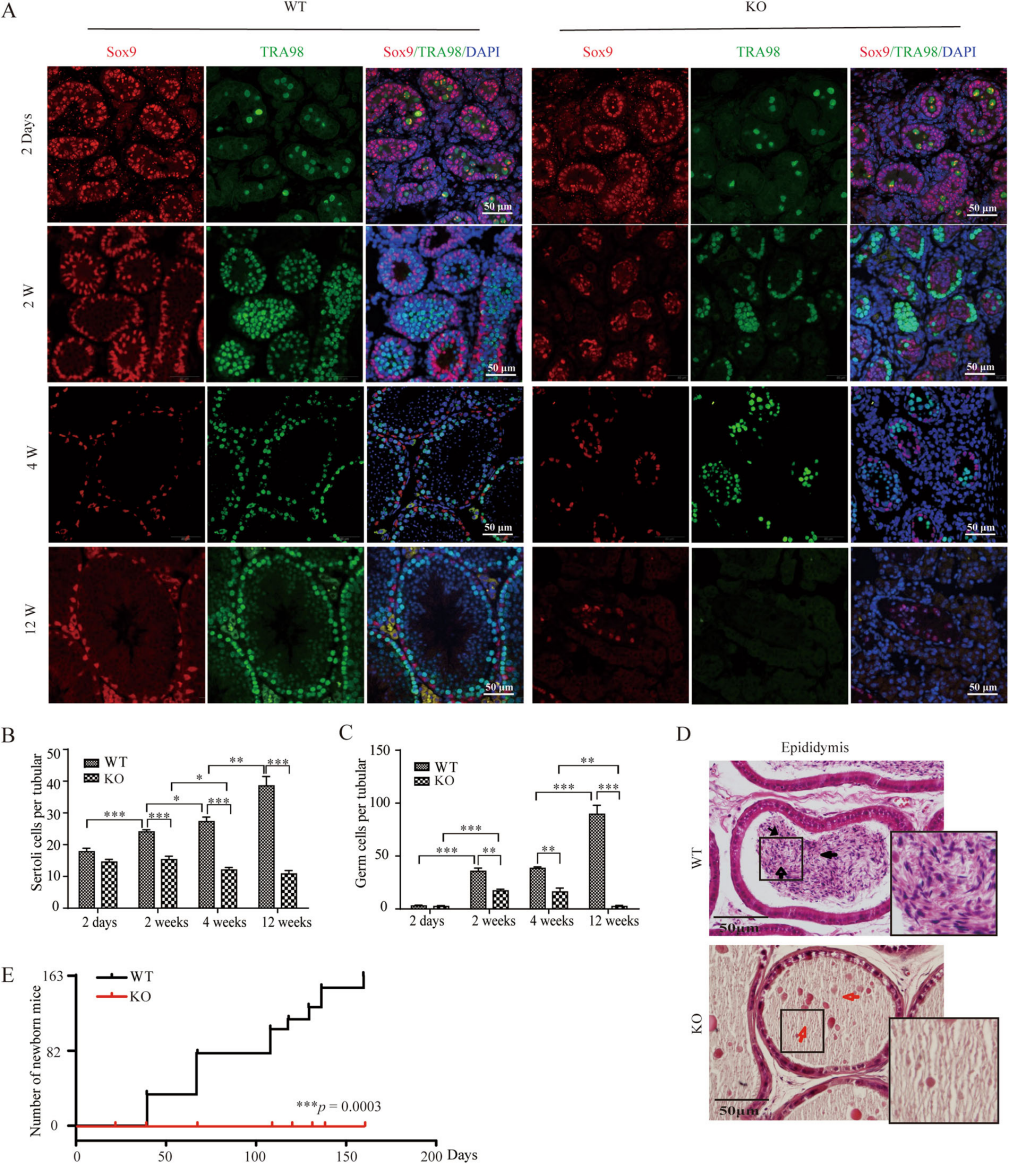
Male mice with Kindlin-2 specific knockout in Sertoli cells are infertile. **A** Immunofluorescence staining for Sox9 (red) or TRA98 (green) in WT and KO testes samples at 2 day, 2-, 4- and 12-W old. The nuclei were stained blue with DAPI. KO mice showed a phenotype corresponding to an age-dependent loss of GCs in seminiferous tubules. By 12 W, KO seminiferous tubules are filled only with Sox9 positive SCs, whereas completely loss of GCs. Scale bar, 50 μm. **B** Quantitation of Sox9 positive SCs per tubule in testes of the WT and KO mice at indicated age point, 6 tubules were counted per section. The data were expressed as the mean ± S.D. Statistical analyses were performed using Student’s *t*-test at indicated time point. The variance was similar between the groups. *p < 0.05, **p < 0.01, ***p < 0.001. **C** Quantitation of TRA98 positive GCs per tubule in testes of the WT and KO mice at indicated age point, 6 tubules were counted per section. The data were expressed as the mean ± S.D. Statistical analyses were performed using Student’s *t*-test at indicated time point. The variance was similar between the groups. *p < 0.05, **p < 0.01, ***p < 0.001. **D** HE staining of epididymis of 8 W WT and KO mice. The epididymis of WT mice was filled with mature sperms (black arrows). However, no spermatocytes or mature sperms were detectable in epididymis of KO mice (red arrow). Scale bar, 50 μm. **E** Fertility test that KO males crossed to WT females was detected. Reproductive ability of the KO line was tracked over 160 days breeding period. Fertility test were showed by percent of pregnant females. *n* = 6 for KO or WT males. ***p = 0.0003.

To investigate the reproductive capacity of KO mice, IF analysis was performed to evaluate the number and distribution of SCs (Sox9) and GCs (TRA98) at 2 days and 2, 4, and 12 W after birth (Fig. 2A). The results indicated that at day 2, the cord size and the numbers of SCs and GCs in the Kindlin-2 KO testis were not significantly different from WT controls, corresponding to the results of hematoxylin and eosin (H&E) staining (Supplementary Fig. 2). Obvious damage could be observed in the KO testis from 2 W. A small number of residual GCs were clustered together and it was not possible to determine the stages of spermatogenic cells at 4 W (Fig. 2A, Supplementary Fig. 2B). Quantitative analysis showed that the number of SCs and GCs in KO group did not increase, but decreased significantly at 3 months. (Fig. 2B, C). As shown in Fig. 2D, the KO mice had completely empty epididymal tubules (red arrows), without any mature spermatozoa (black arrows). Based on the markedly aberrant testis size and impaired spermatogenesis in KO mice, we hypothesized that these mice would be infertile. Therefore, the reproductive ability of male KO mice was assessed by mating adult male WT or KO mice with adult WT females (*n* = 6 mating pairs) for up to 160 days. In accordance with our expectations, females mated with KO males were completely infertile (*p* = 0.0003) and no litters were obtained even though vaginal plugs were observed after mating (Fig. 2E), while WT mice gave rise to 163 pups. Taken together, these observations strongly suggested that Kindlin-2 is essential for maintaining the SC and GC populations in the seminiferous tubules and for male fertility.

### Loss of Kindlin-2 impaired the biological functions of Sertoli cells

**Fig. 3:**
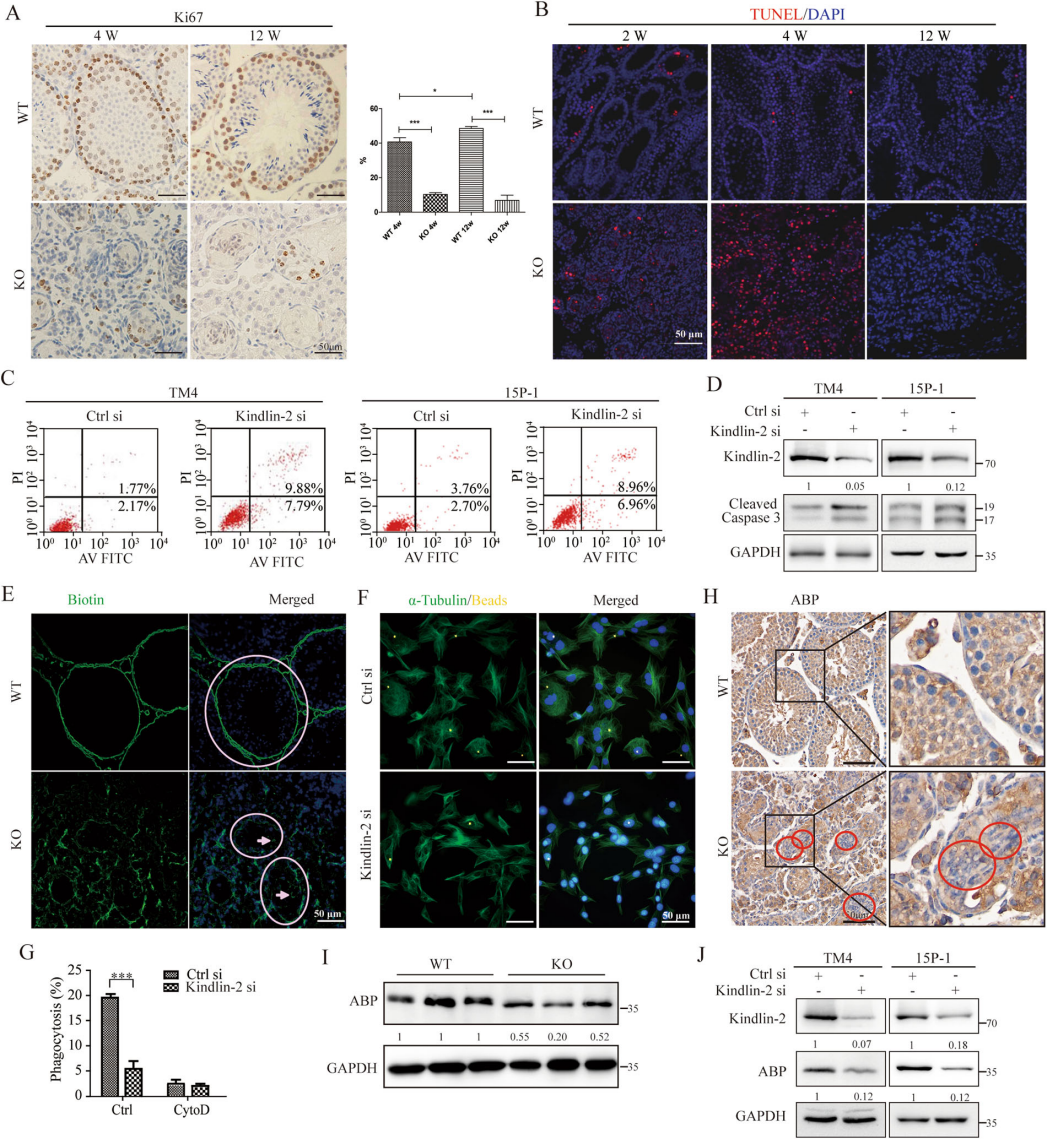
Depletion of Kindlin-2 increases apoptosis and inhibits phagocytosis in Sertoli cells. **A** Left: Ki67 was detected in 4 and 8 W WT and KO testes by immunohistochemical analyses. Scale bar, 50 μm. Right: The quantification of Ki67 positive cells in one Seminiferous tubule. Data are mean ± s.e.m. The variance was similar between the groups. *p < 0.05, **p < 0.01 and ***p < 0.001 by Student’s *t*-test. **B** TUNEL detection for apoptosis (red) in WT and KO testes samples at 2, 4 and 12 W. The nuclei were stained blue with DAPI. KO testes showed increased apoptosis signaling at 2 W testes compared with WT testes. Scale bar, 50 μm. **C** Flow cytometry analysis were used to analyze the apoptosis rate in TM4 and 15P-1, transfected with control or Kindlin-2 siRNA separately. Knock down of Kindlin-2 induced more apoptosis in both TM4 and 15P-1 cells. **D** The Sertoli cell lines TM4 and 15P-1 were transfected with control or Kindlin-2 siRNA for 48 h. Cells were lysed and immunoblotted for Cleaved Caspase 3. β-actin was used as a loading control. Experiments were repeated three times. **E** Immunofluorescence staining of EZ-link sulfo-NHS-LC-biotin tracer was performed in wild type and KO mice testis. WT mice show restriction of biotin tracer to the basal compartment, while KO mice show spreading of biotin tracer dye in seminiferous tubules. Scale bar, 50 μm. Circles indicate the seminiferous tubules, and arrows showed the biotin entering seminiferous tubules through blood testes barrier. **F** Immunofluorescence of α-tubulin (green) was performed 3 h after the microbeads phagocytosis test in Kindlin-2 siRNA compared with control siRNA in 15P-1 cells. Scale bar, 50 μm. **G** Quantification for the phagocytosis ratio in control and Kindlin-2 siRNA in 15P-1 cells. Cells treated with Cytochalasin D were used as negative control. Statistical analyses were performed using Student’s *t*-test. The variance was similar between the groups. ***p < 0.001. **H** Immunohistochemistry staining for ABP in testes from 4 W WT and KO mice testis. Scale bar, 50 μm. Magnifications were shown on the right panel. **I** Western blot analyses of ABP in testes from 4 W KO and WT mice. GADPH acts as an internal reference. **J** Representative western blot analyses of ABP and Kindlin-2 using specific antibodies through loss of function of Kindlin-2 in two cell lines TM4 and 15P-1. GADPH acts as an internal reference.

As shown in Fig. 3A, IHC analyses showed that the number of Ki67-positive cells in seminiferous tubules was significantly decreased in Kindlin-2 KO mice at 4 W compared to WT mice, while the number of terminal deoxynucleotidyl transferase dUTP nick end labeling (TUNEL)-positive cells in seminiferous tubules was significantly higher than in WT mice at 2 and 4 W (Fig. 3B). Furthermore, Kindlin-2 depletion was shown to increase the ratio of apoptotic cells (Fig. 3C) by upregulating cleaved caspase-3 in the SC lines TM4 and 15P-1 (Fig. 3D). As shown in Fig. 3E, the biotin tracer could not penetrate the BTB and was restricted outside of the basal compartment in WT mice. However, in the KO testis, biotin tracer dye spread into the seminiferous tubules, suggesting that loss of Kindlin-2 leads to a leaky BTB structure and abnormal function (Fig. 3E). The results of the in vitro assay indicated that the phagocytosis ability of 15P-1 cells was markedly reduced when Kindlin-2 expression was knocked down, which was similar to cells treated with the phagocytosis inhibitor, cytochalasin D (Cyto D)^17^ (Fig. 3F). ABP was also shown to be reduced in the testes of KO mice (Fig. 3H, I) and Kindlin-2-depleted SC lines, in comparison to controls (Fig. 3J). Taken together, these results indicated that Kindlin-2 reduction disrupted the important biological functions of SCs, including proliferation, survival, barrier function, phagocytosis, and important protein synthesis.

### Loss of Kindlin-2 increases YAP phosphorylation and inhibits YAP nuclear translocation

**Fig. 4:**
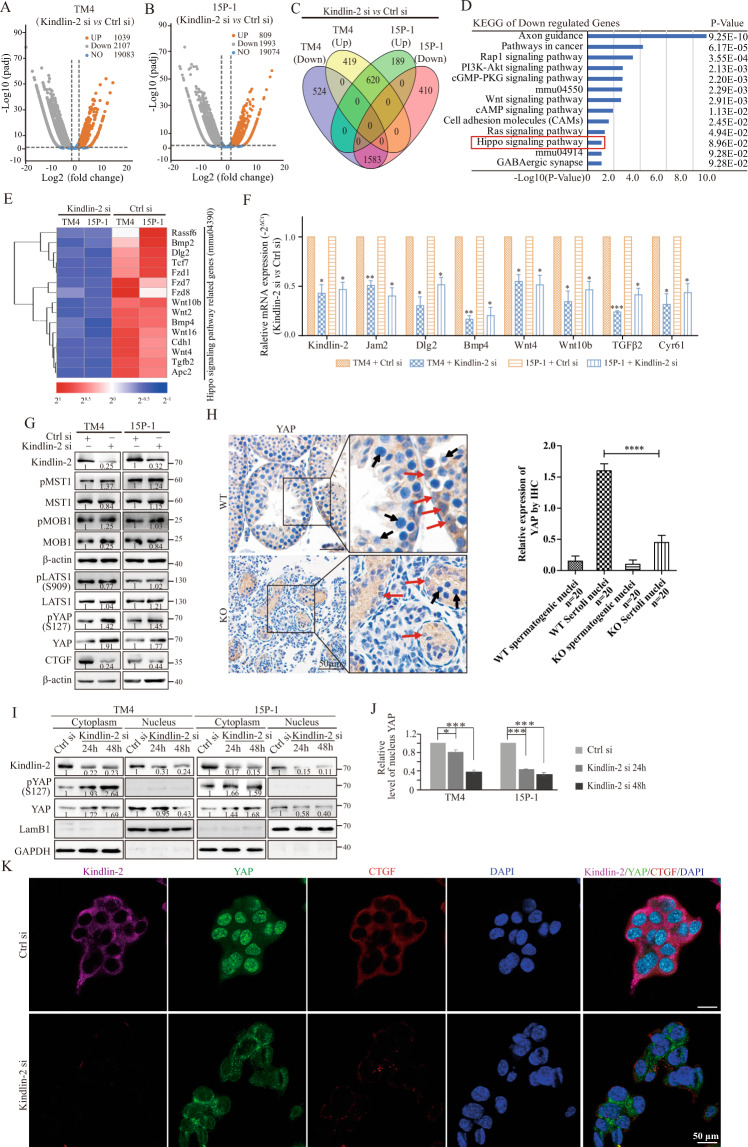
Depletion of Kindlin-2 activates Hippo pathway and inhibits YAP nuclear localization. **A, B** Volcano plots showing differential expression in TM4 (**A**) or 15P-1 (**B**) cells transfected with Kindlin-2 siRNA or control siRNA. Genes which have greater than or equal twice-fold difference expression among two groups were shown in gray (downregulated) or orange (upregulated), *P*_adj_ < 0.05. **C** Overlap of genes regulated by depletion of Kindlin-2 in TM4 (**A**) and 15P-1 (**B**) cells were shown in Venn diagram. Comparing transcript abundances in control cells and Kindlin-2 inhibited cells revealed 3146 and 2802 significantly altering genes in TM4 and 15P-1 cell respectively. A total of 2203 altering genes overlapped in both two cells, in which 1583 genes were down-regulated and 620 genes were up-regulated. *P*_adj_ < 0.05. **D** KEGG enrichment of 1583 down-regulated genes after Kindlin-2 depletion. KEGG analysis was carried out by using DAVID online tool. Enrichment scores were shown as −log10 (*P*-value). **E** A heat map analysis of Hippo signaling pathway related genes. Blue and red square frames represent low or high expression levels respectively. **F** Control and Kindlin-2 siRNAs were transiently transfected into TM4 and 15P-1 cells separately, after 48 h, qPCR was performed to detect the expression of cell junction related genes (*Jam2* and *Dlg2*) and Hippo signaling genes (*Wnt4, Wnt10b, TGFβ2* and *Cypr61*) using specific primers. GAPDH was used as internal reference. Three independent experiments were involved. Data are mean ± s.e.m. The variance was similar between the groups. *p < 0.05, **p < 0.01 and ***p < 0.001 by Student’s *t*-test. **G** Representative Western blot analyses of Hippo signaling pathway factors pMST1, MST1, pMOB1, MOB1, pLATS1-909, LATS1, pYAP-127 and YAP using specific antibodies through loss of function of Kindlin-2 in two cell lines TM4 and 15P-1. β-actin acts as an internal reference. **H** Left: Representative immunohistochemistry detection for YAP in 4 W WT and KO mice testis. The red arrows show SCs nucleus and the black arrows showed the nucleuses of spermatogenic cells. Scale bar, 50 μm. Magnifications were shown on the right panel; red arrows indicate the staining of YAP in Sertoli cells. Right: quantitative analysis to compare the amount of Yap in spermatogenic nuclei and Sertoli nuclei of KO testes to WT testes. The variance was similar between the groups. ***p < 0.001 by Student’s *t*-test. **I** Nuclear and cytoplasmic proteins isolated from TM4 and 15P-1 cells transfected with control or Kindlin-2 siRNAs were subjected to immunoblot analysis using antibodies against Kindlin-2, YAP and pYAP (S127). LamB1 and GAPDH were used as loading controls for nucleus and cytoplasm respectively. **J** Quantifications of nuclear YAP of two Sertoli cell lines upon Kindlin-2 silencing or not. **K** Confocal images of Kindlin-2 (purple), YAP (green), CTGF (red) and DAPI (blue) immunofluorescence staining of 15P-1 Ctrl si cells and 15P-1 Kindlin-2 si cells (transient transfection). Scale bar, 50 µm.

To determine the molecular mechanism underlying the pivotal role of Kindlin-2 in SCs, the gene expression profiles in Sertoli cell lines TM4 and 15P-1 with control or Kindlin-2 siRNA were examined by RNA-Seq assay (Fig. 4A, B). Overlapping genes upregulated or downregulated by Kindlin-2 (fold change > 2) are shown in Fig. 4C. Furthermore, enriched biological pathways involving the above-mentioned genes were analyzed using the Database for Annotation, Visualization and Integrated Discovery (DAVID). The differentially expressed genes were associated with the development of urogenital and renal systems, as well as organ morphogenesis and embryonic organ development (Supplementary Fig. 3A, B). In addition, heatmap analysis of the cell-cell junction term (GO0005911) revealed that the expression levels of cell junction and cell polarity-related genes, including *Ocln*, *Jam2*, *Cldn1*, *Cdh1*, and *Pard3b*, were significantly downregulated due to Kindlin-2 depletion (Supplementary Fig. 3C), consistent with the observed Kindlin-2 depletion-related BTB impairment. KEGG enrichment analysis revealed that a number of genes downregulated by Kindlin-2 were associated with various signaling cascades, including the Rap1, PI3K-Akt, and Wnt pathways (Fig. 4D), consistent with the known signaling pathways regulated by Kindlin-2^18,19^. The Hippo signaling pathway (Fig. 4D, red) attracted our attention as it is known to be crucial for regulation of cell growth and organ size^20,21^. Genes related to the Hippo signaling pathway identified by KEGG enrichment analysis is shown in Fig. 4E. RT-qPCR analyses were performed to validate these genes in the cultured SC lines TM4 and 15P-1. Transient transfection of Kindlin-2 or control siRNAs into TM4 and 15P-1 cells indicated that loss of Kindlin-2 downregulated Hippo pathway-related genes, including *Bmp4*, *Wnt4*, *Wnt10b*, and *TGFβ2*. The expression levels of the cell polarity protein *Dlg2* and the junction protein *Jam2* were also significantly decreased in Kindlin-2-depleted SCs. Remarkably, the expression of *Cyr61*, a downstream transcriptional target of YAP, was also downregulated in Kindlin-2-depleted SCs (Fig. 4F). The above data suggested that testis dysgenesis may be mediated by Kindlin-2 depletion-induced Hippo signaling pathway activation. Western blotting analysis confirmed that depletion of Kindlin-2 increased the levels of YAP and pYAP-S127, and decreased the level of connective tissue growth factor (CTGF; a target gene of YAP) (Fig. 4G).

Next, IHC analysis was performed to examine the level and localization of YAP in mouse testicular tissues, and the results indicated that loss of Kindlin-2 in SCs decreased the nuclear localization of YAP (Fig. 4H, left). To further clarify this change, quantitative analysis was performed to compare the levels of Yap in spermatogenic and SC nuclei in the testes of KO and WT mice (Fig. 4H, right). YAP protein levels in the cytoplasm and nucleus were evaluated biochemically in relation to Kindlin-2 depletion at different time points. The results showed that depletion of Kindlin-2 led to a decrease in the level of YAP in the nuclear extract and an increase in YAP in the cytoplasmic fraction in both TM4 and 15P-1 cells (Fig. 4I, J). Consistent with these observations, IF analysis indicated that Kindlin-2 depletion in 15P-1 cells decreased the level of YAP in the nuclei, with a corresponding decrease in the level of CTGF (Fig. 4K). Taken together, these findings indicated that Kindlin-2 regulates YAP phosphorylation and nuclear translocation through a mechanism that does not involve alterations of the kinase activity of LATS1 in SC cells.

### Kindlin-2 binds to the kinase domain of LATS1 and intercepts the interaction between LATS1 and YAP

**Fig. 5:**
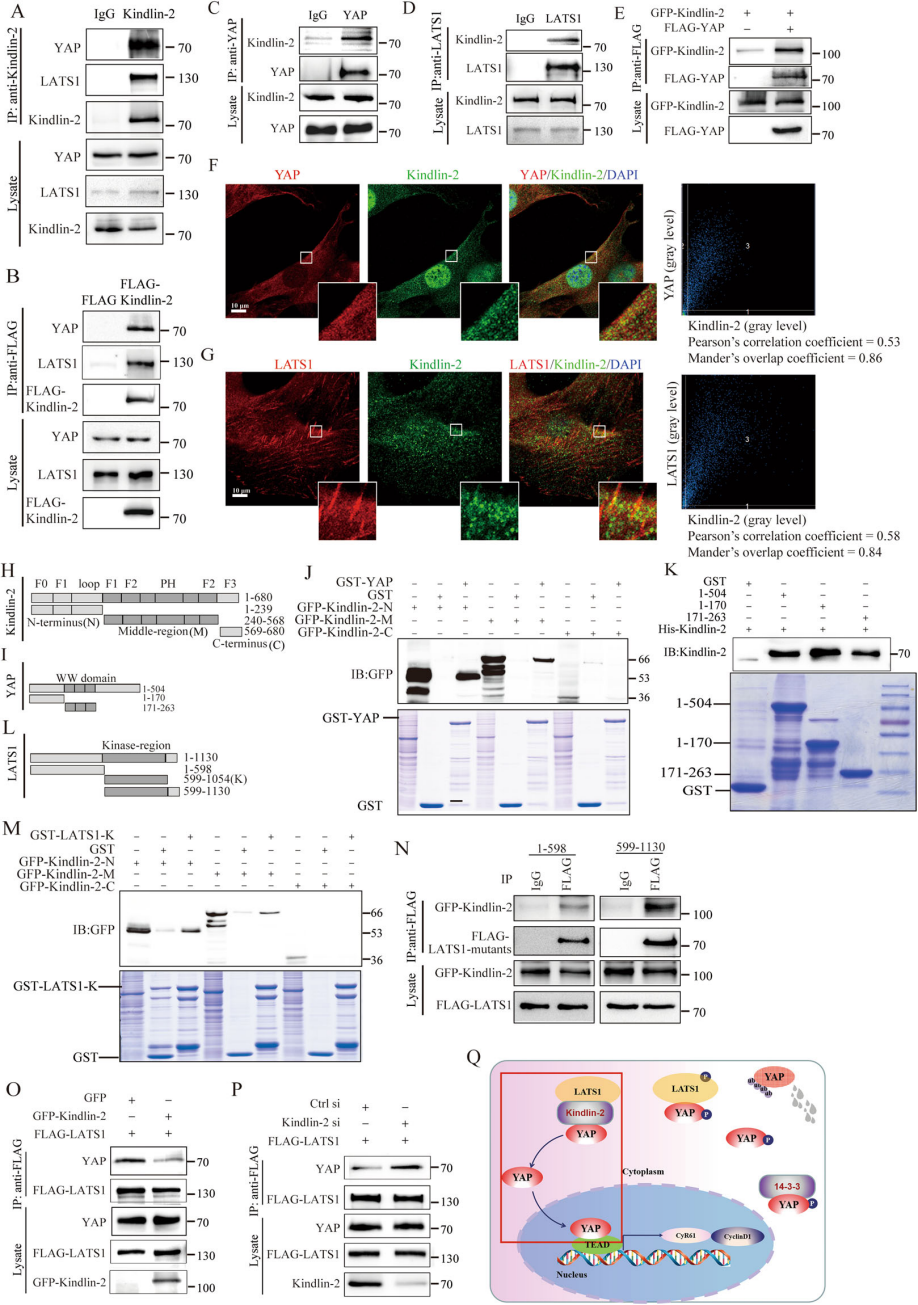
Kindlin-2 interacts with both LATS1 and YAP and impedes the interaction between LATS1 and YAP. **A** Total lysates were extracted from TM4 cell lines (without treatments) for co-immunoprecipitation (Co-IP) experiments. Equal amount of proteins was immunoprecipitated separately with IgG or anti-Kindlin-2 antibody. Western blot displayed the expression of YAP, LATS1 and Kindlin-2 with specific antibodies. **B** FLAG and FLAG-Kindlin-2 expression vector were transfected into TM4 cell lines separately. 48 h after transfection, total cell lysates were immunoprecipitated with anti-FLAG antibody and protein G-Sepharose. The expression of YAP, LATS1, and FLAG were detected with specific antibodies by Western blot analysis. **C** Anti-YAP antibody was used for Co-IP experiment and the expression of Kindlin-2 was detected by specific antibodies. **D** Anti-LATS1 antibody was used for Co-IP experiment and the expression of Kindlin-2 was detected by specific antibody. **E** GFP-Kindlin-2 and FLAG-YAP expression vector were transfected into HEK293T cell lines separately. 48 h after transfection, total cell lysates were immunoprecipitated with anti-FLAG antibody and protein G-Sepharose. The expression of YAP and FLAG were detected with specific antibodies by Western blot analysis. **F** 15P-1 cells were stained with an anti-YAP mouse (red) and an anti-Kindlin-2 rabbit (green). Nuclei were stained with DAPI (blue), followed by visualization with confocal microscopy. Scale bars, 10μm. Magnification were shown. **G** 15P-1 cells were stained with an anti-LATS1 mouse (red) and an anti-Kindlin-2 rabbit (green). Nuclei were stained with DAPI (blue), followed by visualization with confocal microscopy. Scale bars, 10 μm. Magnification were shown. **H**–**J** Indicated truncates of Kindlin-2, YAP and LATS1 were constructed according to the functional domains respectively. YAP contains two WW domains in the middle and a transactivation domain at the C terminus. LATS1 contains a kinase region in the middle part. **K** HEK293 cells were transfected with the indicated truncates of GFP-Kindlin-2. Cell lysates were incubated with GST or GST-YAP in vitro for GST pull-down assays followed by immunoblotting using an anti-GFP antibody. **L** Purified His-Kindlin-2 protein was incubated with purified GST or GST-YAP fragments at 4 °C overnight. Beads were washed and the remaining proteins were resolved by SDS-PAGE and further analyzed by Western blot analysis using anti-Kindlin-2 antibody (upper panel). The GST and GST-YAP were stained by Coomassie blue (lower panel). **M** HEK293 cells were transfected with the indicated truncates of GFP-Kindlin-2. Cell lysates were incubated with GST or GST-LATS1-Kinase region in vitro for GST pull-down assays followed by immunoblotting using an anti-GFP antibody. **N** HEK293 cells were co-transfected with GFP-Kindin-2 and the indicated truncates of FLAG-LATS1. Cell lysates were incubated with anti-FLAG antibody and protein G-Sepharose. The expression of GFP and FLAG were detected with specific antibodies by Western blot analysis. **O**, **P** Kindlin-2 isolates the interaction between LATS1 and YAP. **O** GFP or GFP-Kindlin-2 were co-transfected with FLAG-LATS1 into HEK293T cell lines. **P** Control siRNA or Kindlin-2 siRNA were co-transfected with FLAG-LATS1 into HEK293T cell lines. Co-immunoprecipitation was performed after 48 h transfection with FLAG-M2 beads. Western blot showed the expression of immunoprecipitation lysis YAP and FLAG and unprocessed lysis YAP, FLAG and Kindlin-2. **Q** Within the red rectangle is the brief working model of Kindlin-2 inactivating Hippo signaling pathway by interacting with LATS1 and YAP to inhibit the phosphorylation of YAP and to promote YAP nuclear localization. Outside the red rectangle is widely known Hippo signaling activation process.

To elucidate this mechanism, we first examined the associations of Kindlin-2 with the key components of the Hippo pathway in SCs. The results showed that both endogenous Kindlin-2 and FLAG-Kindlin-2 co-immunoprecipitated endogenous YAP and LATS1 in living TM4 cells (Fig. 5A, B). Endogenous YAP and endogenous LATS1 also co-immunoprecipitated endogenous Kindlin-2 in HEK293T cells (Fig. 5C, D). Furthermore, GFP-Kindlin-2 was co-immunoprecipitated by FLAG-YAP in HEK293T cells (Fig. 5E). Moreover, IF analysis showed that Kindlin-2 co-localized with YAP and LATS1 in TM4 cells (Fig. 5F, G). These results indicated that Kindlin-2 interacts with LATS1 and YAP in living cells, i.e., Kindlin-2 is able to form a molecular complex with LATS1 and YAP.

To identify the key regions of Kindlin-2 involved in binding to YAP or LATS1, we constructed a series of mutants proteins (Fig. 5H, I, L). GST pull-down assays showed that, full-length YAP interacted with the N-terminal and middle regions of Kindlin-2 (Fig. 5J) and the Kindlin-2 directly interacted with both the N-terminal and WW domains of YAP (Fig. 5K). LATS1 kinase domain interacted with the N-terminal and middle regions of Kindlin-2 (Fig. 5M). In addition, the full-length Kindlin-2 was also found to interact strongly with the kinase domain of LATS1 in a co-immunoprecipitation assay in living cells (Fig. 5N).

As LATS1 also binds to the WW domain of YAP through the PPPY^559^ motif^22,23^, the ability of YAP to bind LATS1 was examined in the presence of altered levels of Kindlin-2 in cells co-transfected with FLAG-LATS1. The results showed that ectopic expression of Kindlin-2 inhibited LATS1 binding to YAP (Fig. 5O), while Kindlin-2 depletion enhanced the interaction of LATS1 with YAP (Fig. 5P). These findings indicated that Kindlin-2 intercepts the LATS1-YAP interaction. A model illustrating their interactions is shown in Fig. 5Q.

### Loss of Kindlin-2 is correlated with Sertoli cell-only syndrome (SCOS) in men

**Fig. 6:**
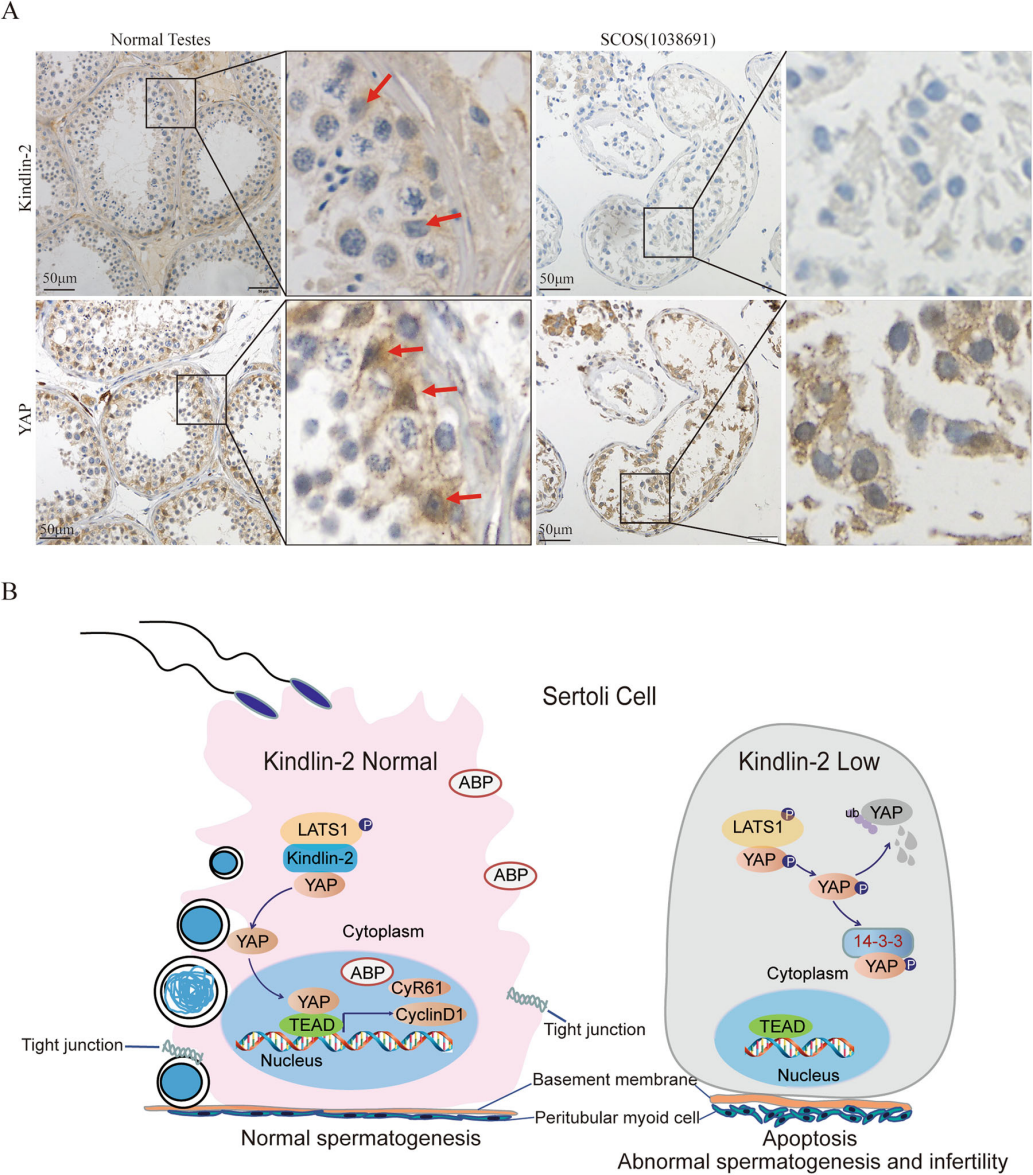
Expression of Kindlin-2 and YAP in human SCOS and role of Kindlin-2 in Sertoli cell. **A** Representative IHC staining of Kindlin-2 and YAP expression in normal human testes and SCOS patient specimens. Scale bar, 50 μm. Local-magnification is on the right panel of each pictures respectively. **B** Working model for the role of Kindlin-2 in Hippo/YAP signaling regulation and Sertoli cell functions. Loss of Kindlin-2 in Sertoli cells mediating YAP activation in SCs growth, and leads to change of biological function.

IHC analyses were performed using testis sections from 16 SCOS patients and three normal men. Kindlin-2 was expressed in both the cytoplasm and nucleus of SCs, and YAP was mainly localized in the nucleus of normal human SCs (Fig. 6A). The IHC results revealed that 5 of 16 SCOS samples showed decreased Kindlin-2 expression in SCs. In addition, 13 of 16 SCOS samples showed loss of YAP nuclear localization, consistent with the observations in SC-specific Kindlin-2 KO mice. These data suggested that SOCS patients have loss of Kindlin-2 and subsequent Hippo signaling pathway activation.

## Discussion

It is estimated that about 5–10% of infertile men may have SCOS, the only symptom of which is infertility^7^. Histologically, patients with SCOS lack spermatogenic cells in almost all seminiferous tubules^6^. In the present study, we identified a novel mechanism underlying male infertility in a mouse model with knockout of Kindlin-2, which controls testicle development and spermatogenesis. Depletion of Kindlin-2 in SCs results in suppression of Hippo signaling and leads to phosphorylation and cytoplasmic retention of YAP. Less or no YAP would undergo translocation into the nucleus, leading to suppression of the transcriptional activation of downstream target genes. Meanwhile, loss of Kindlin-2 inhibits the expression of genes related to cell growth and cell-cell junctions, resulting in abnormal testis development, impaired spermatogenesis, and infertility in male mice (Fig. 6B).

SCs are nurse cells that provide support for spermatogenesis, constituting the basis of male reproductive function^24^. In this study, loss of Kindlin-2 in SCs was shown to impact cell proliferation in seminiferous tubules and cause depletion of GCs and SCs by apoptosis, finally leading to shrinkage of the seminiferous tubules. Our previous studies also demonstrated that Kindlin-2 regulates cell adhesion and apoptosis^25,26^. The relative lack of apoptotic cells in the seminiferous tubules in the 12-week KO group may have been because significant apoptosis had already caused a marked decrease in the number of cells in the tubules at this time point. In addition, Kindlin-2 knockout caused collapse of the architecture of seminiferous tubules, including impairment of cell-cell junctions and the structure of the BTB. In addition, to maintain the integrity of SCs to support spermatogenesis, we also showed that Kindlin-2 regulates the level of ABP expression in SCs and the phagocytotic ability of SCs. Our results demonstrated that Kindlin-2 is a pivotal molecule in maintenance of the structure and biological functions of SCs. The structural and functional abnormalities contributed to small testis and infertility in Kindlin-2 knockout mice.

This study focused on the role of Kindlin-2 in regulation of the structure and function of SCs during testis development. RNA-Seq analysis of SCs revealed a new mechanism by which Kindlin-2 modulates activation of Hippo signaling, and finally regulates SC proliferation.

In most species, SCs proliferate in fetal or neonatal life and in the peripubertal period, which determines the final number of SCs in adulthood^10^. Levasseur et al. reported that conditional knockout of YAP and TAZ in SCs led to an increase in apoptotic cells in neonates and a decrease in SCs in the testis of adult mice^27^. However, the latter study did not elucidate the functions of YAP and TAZ in SCs. We demonstrated previously that Kindlin-2 played a critical role in regulation of the Hippo/YAP signaling pathway by complexing with LATS1 and MOB1 in kidney cells^28^. In this study, as a novel mechanism of modulation of YAP phosphorylation, we showed that Kindlin-2 isolates LATS1 from closing to the WW domain of YAP and impedes binding of the LATS1 kinase domain to YAP (Fig. 5Q). Loss of Kindlin-2 increased YAP phosphorylation and caused its retention in the cytoplasm, thereby suppressing Hippo/YAP signaling and inhibiting organ growth and development of the testis. Here we found that loss of Kindlin-2 increased the level of YAP in the SC lines TM4 and 15P-1, and that the regulation of YAP phosphorylation was dependent on LATS1 activation mediated by Kindlin-2. Kindlin-2 interacts with both LATS1 and YAP. Interestingly, Kindlin-2 is situated between LATS1 and YAP, spatially separating LATS1 from YAP, preventing LATS1 from accessing and phosphorylating YAP. Therefore, YAP could not be activated, and non-phosphorylated YAP would be translocated into the nucleus where it would activate target gene transcription together with TEA domain family members (TEADs). Therefore, our findings were distinct from those of Guo et al. in MSCs^29^ and of Song et al. in kidney cells^28^. Our study suggested that the regulatory effect of Kindlin-2 on Hippo signaling may vary in different cell types. These discrepancies in comparison with previous reports suggested that regulation of the Hippo signaling pathway by Kindlin-2 may be cell type-specific, and further studies are required to elucidate the detailed molecular mechanisms underlying the modulation of Hippo signaling by Kindlin-2.

Kindlin-2 maintains cell-cell junctions in SCs. It is known that cell-cell junctions, cell polarity, and the cytoskeleton regulate Hippo signaling^30^. The best example of this is provided by junctional proteins; when cells reach a high density, these proteins transmit growth inhibitory signals by regulating Hippo/YAP signaling, making them ideal tools to monitor the extracellular environment. Studies in *Drosophila*, zebrafish, and mammalian cells (e.g., cancer cells) have also revealed that many cell polarity proteins are upstream regulators of the Hippo/YAP pathway^30^. In this study, we showed that Kindlin-2 depletion in SCs induced collapse of cell-cell junctions and inhibition of cell proliferation, which may represent an alternative mechanism of regulation of Hippo/YAP signaling during testicle development.

In summary, the present study identified a new mechanism underlying male infertility. Kindlin-2 is essential for maintaining the structure and function of SCs in the seminiferous tubules of the testis. Specific depletion of Kindlin-2 in SCs of the mouse testis led to SC apoptosis and germ cell depletion, resulting in damage to the seminiferous tubules in the testis and complete infertility in male mice, resembling the clinical pathology of SCOS patients. This study suggested that Kindlin-2 depletion may be responsible for male infertility, especially SCOS. In addition, we developed a mouse model of SCOS that could be useful in future investigations.

## Materials and methods

### Ethics statement

All animal experiments were approved by the Peking University Biomedical Ethics Committee and the approval number is: LA2018295. Sertoli Cell Only Syndrome testis and normal testis are clinical case test samples with informed consent from Peking University third hospital, and was approved by Medical Ethics committee of Peking University Third Hospital.

#### Reagents and antibodies

Primary antibodies were used at the indicated concentrations for Western blotting (WB), IF, or IHC analyses as follows: WT1 (SC192; Santa Cruz); StAR (8449; Cell Signaling); TRA98 (ab82527; Abcam); Caspase-3 (9662; Cell Signaling); Ki67 (AF7649; RD system); rabbit anti-Kindlin-2 (K3269; Sigma-Aldrich, St. Louis, MO, USA): WB, 1:1000; IHC, 1:200; mouse anti-Kindlin-2 (clone 3A3; Mab2617; EMD Millipore, Billerica, MA, USA): WB, 1:1000; IF, 1:200; mouse anti-FLAG (clone M2; Sigma-Aldrich): WB, 1:2000; mouse anti-GFP (clone GSN149; G1546; Sigma-Aldrich): WB, 1:2000; mouse anti-actin (clone 2Q1055; sc-58673; Santa Cruz Biotechnology, Inc., Dallas, TX, USA): WB, 1:2000; rabbit anti-LATS1 (C66B5; # 3477; Cell Signaling Technology): WB, 1:1000; IHC, 1:200; rabbit anti-pLATS1-S909 (#9157; Cell Signaling Technology): WB, 1:1000; IHC, 1:200; rabbit anti-YAP (D8H1X; 14074 S; Cell Signaling Technology): WB, 1:1000; IF, 1:50; IHC, 1:200; rabbit anti-pYAP-S127 (13008 s; Cell Signaling Technology): WB, 1:1000; IHC, 1:200; mouse anti-GAPDH (TA-08; Zhong Shan Jin Qiao, Beijing, China): WB, 1:1000.

Secondary antibodies included Donkey anti-Rabbit Alexa Fluor 488 (Invitrogen; Cat# CA21202S); Donkey anti-Goat Alexa Fluor 568 (Invitrogen; Cat# SC28737); Goat anti–Rat IgM Alexa Fluor 488 (Invitrogen; Cat# A21248). Goat anti-mouse horseradish peroxidase (HRP) and goat anti-rabbit HRP (both Santa Cruz Biotechnology, Inc.): WB, 1:4000. Proteasome inhibitor MG132 (SML1135) and protein synthesis inhibitor cycloheximide (C7698) were purchased from Sigma-Aldrich.

Critical Commercial Assays included Annexin V FITC Apop Dtec Kit I (BD; Cat# 556547); HChamQ SYBR qPCR Master Mix (Vazyme; Cat# Q331); HiScript II Q RT SuperMix for qPCR (Vazyme; Cat# R223); TUNEL BrightRed Apoptosis Detection Kit (Vazyme; Cat# A113); SUPERSIGNAL WEST PICO CHEM (Thermo Scientific; Cat# 34080); PER NUCLEAR AND CYTOPLASMIC EXTRACTION (Thermo Scientific; Cat# 78833).

#### Generation of mice

To generate Sertoli cells specific conditional Kindlin-2 knockout mice, the *Kindlin-2*^*f/f*^ mice and Amh-Cre mice were crossed to obtain *Amh-Cre*; *Kindlin-2*^*f/f*^ as experimental group. The floxed *Kindlin-2* (*Kindlin-2*^*f/f*^) mouse strain, which carries loxP sites flanking exon 5 and 6, were purchased from the International Knockout Mouse Consortium (IKMC). The *Kindlin-2*^*f/f*^ mice crossed with Amh-Cre mice to generate *Amh-Cre*; *Kindlin-2*^*f/+*^ mice, Then the *Amh-Cre*; *Kindlin-2*^*f/+*^ mice were intercrossed to generate *Amh-Cre*; *Kindlin-2*^*f/f*^ mice which referred to as KO mice. For all animal studies transgenic mice were backcrossed eight times to C57BL/6. After the animal has been identified, animal observation of each genotype is randomized.

#### Genotyping

Primers used in PCR genotyping of WT or KO mice were as follows, genotypes were determined by multiplex polymerase chain reaction (PCR) using DNA prepared from mouse tail samples.

*Loxp* forward primer (5′ to 3′): tacaggtggctgacaagatcc;

*Loxp* reverse primer (5′ to 3′): gtgaggctcacctttcagagg;

*Cre* forward primer (5′ to 3′): tccaatttactgaccgtacaccaa;

*Cre* reverse primer (5′ to 3′): cctgtacctggcaatttcggcta.

Primers for genotyping the *Kindlin-2*^*f/+*^ and *Kindlin-2*^*f/f*^ mice were detected using primers LoxP-F and LoxP-R with a 743 and 839 bp PCR product, respectively.

The reaction conditions were: 94 °C for 5 min; 35 cycles of 94 °C for 30 s; 57.5 °C for 30 s; 72 °C for 30 s; final extension step of 72 °C for 5 min.

PCR genotyping of Cre mice using primers Cre-F and Cre-R with following conditions: 94 °C for 5 min; 35 cycles of 94 °C for 30 s; 61.5 °C for 30 s; 72 °C for 30 s.

#### Histological immunostaining

The testes or epididymis tissues were fixed in Bouin’s solution for hematoxylin and eosin (HE) staining or in 4% formaldehyde (PFA) in PBS for immunostaining. In brief, tissues were fixed overnight, embedded in paraffin wax, and cut to produce 5 μm-thick sections. For immunohistochemistry (IHC), the sections were dewaxed in xylene and rehydrated in serial dilutions of alcohol. Endogenous peroxidase was blocked by immersing the sections in 0.3% H_2_O_2_ in methanol for 20 min at room temperature. The sections were then blocked with 5% bovine serum albumin (BSA) and incubated with the primary antibody at 4 °C overnight, and then the secondary antibody was applied for 1 h. Staining was visualized using a DAB substrate kit according to the manufacturer’s protocol (Zhongshan Technology, Beijing, China). The negative controls were subjected to the same protocol except that PBS was used instead of the primary antibody. We use double blind strategy in tissue analysis experiment.

#### Immunofluorescence and confocal analysis

Cells were seeded and cultured on sterile glass cover slips in six-well plates. 24 h later, cells were fixed in 10 % formaldehyde for 30 min at 37 °C. Cells were permeabilized with 0.2% Triton-X-100 containing 4′,6-diamidino-2-phenylindole for 5 min, and blocked with 2% bovine serum albumin for 1 h. The cells were then stained with specific primary antibodies overnight at 4 °C. After washing, the cells were incubated with secondary antibodies for 1 h at room temperature. Nuclear staining was performed with 40, 6-diamidino-2-phenylindole (DAPI). Cells were then imaged using a confocal microscope (SP2-AOBS Leica Microsystems).

Confocal microscopy was performed using a TCS SP8 (Leica) with a 63 × 1.2 Plan-Apochromat water immersion lens. Software analysis of the converted binary images was performed with Image Processing and Analysis in Java (Image J).

#### Western blot

Protein extracts were fractionated on a 10% SDS–polyacrylamide gel electrophoresis gel and transferred onto polyvinylidene fluoride membranes (Merck Millipore, Billerica, MA, USA). The blots were then blocked with 5% nonfat milk in Tris-buffered saline with Tween 20 (TBST) for one hours at room temperature, after which they were probed with specific primary antibodies, followed by incubation with secondary antibodies conjugated with horseradish peroxidase; and visualized using a Western Blotting Detection Kit (GE Healthcare, cat#: RPN2106).

#### Sertoli cell lines and Cell culture

Sertoli cell lines 15P-1 and TM4 were purchased from Institute of Basic Medical Sciences, Chinese Academy of Medical Sciences (http://www.crcpumc.com/). TM4 cells were cultured at 37 °C with 5% CO_2_ in DMEM/F12 containing 2.5% heat-inactivated fetal bovine serum (FBS) and 5% horse bovine serum (HBS). 15P-1 cells are derived from testicular cells of PyLT transgenic mice, and have properties of Sertoli cells. 15P-1 cells were maintained in DMEM with 10% heat-inactivated FBS and cultured at 32 °C with 5% CO_2_. Cells used in this study were purchased from ATCC. We tested the mycoplasma contamination by PCR during the culture processes and all the cells used here were mycoplasma free.

#### RNA interference

siRNAs against mouse Kindlin-2 and control siRNA (siN0000001-1-10, Ruibo) were purchased from Ruibo Biotechnology. The Kindlin-2 siRNA sequence was as follows: AAGTTGGTGGAAAAACTCGAT. For transient transfection, 60–70% confluent cells were transfected with a total of 10–20 nM siRNA using Lipofectamine RNAiMAX according to the manufacturer’s instructions (Invitrogen). After 8 h, the medium was replaced and cells were further incubated for 48 h for subsequent experiments. The efficacy of Kindlin-2 siRNA knockdown was among 60–80%.

#### Phagocytosis assays

15P-1 cells were seeded on sterile glass cover slips in a 6-well plate and transfected with Kindlin-2 or control siRNA. After 48 h, 1 μl of 2.0-m carboxylate-modified red fluorescent beads (Sigma) in 2 ml medium per well were incubated for 2 h at 32 °C in 5% CO_2_. Phagocytosis assay for Cytochalasin D (Cyto D)-treated cells was performed by transfecting 15P-1 cells followed by Cyto D (C102396, Aladdin) treatment at 50 nM concentration. Cells were fixed and performed immunofluorescence by using α-tubulin primary antibody.

#### Biotin in vivo assay

The permeability of the BTB was assessed by using a biotin tracer. Two-month-old control and Kindlin-2 KO males were anesthetized with avertin and 25 μl of 10 mg/ml EZ-Link Sulfo-NHS-LC-Biotin (Pierce) freshly diluted in PBS containing 1 mM CaCl_2_ was injected into one testis and the other testis was injected with 50 μl of 1 mM CaCl_2_ in PBS as an internal control. The animals were euthanized 30 min later, and the testes were removed, embedded with OCT and immediately frozen. Cryosections were stained with FITC-conjugated streptavidin (1:200) and 1 μg/ml DAPI.

#### TUNEL staining

A TUNEL assay was conducted using an in-situ Cell Death Detection Kit (Promega, CA, USA) as previously described. Briefly, sections of WT and KO testis were prepared and stained using the kit according to the manufacturer’s instruction. Cells were counterstained with hematoxylin. Sections were counterstained with DAPI to identify the nuclei. All positive (red) and negative (blue) nuclei were counted. Apoptotic cells were normalized to the total cells from the same area.

#### Detection of apoptosis

Apoptotic cells were detected using an Annexin V-FITC/PI kit (556547, BD Pharmingen). Briefly, Sertoli cell lines 15P-1 and TM4 were transfected with siRNA as described previously. After 48 h later, cells were harvested and washed with cold PBS twice, and then resuspend cells in Binding Buffer at a concentration of 10^6^ cells/ml. Transfer 100 μl of the solution to a 5 ml culture tube and add 5 μl of FITC Annexin V, gently vortex the cells and incubate for 15 min at 25 °C in the dark. Then add 400 μl of Binding buffer and 5 μl PI to each tube before flow cytometry. Three independent experiments were performed for each analysis.

#### RNA-sequencing and analysis

To identify Kindlin-2 regulated genes, Sertoli cell lines TM4 and 15P-1 were transfected with either Kindlin-2 or Control SiRNA. After 48 h, total RNAs were isolated using the Trizol reagent (Invitrogen) following the manufacturer’s protocol. RNA quality was inspected using Nanodrop (all samples had 260/280-ratio≥1.9). The inhibit rat of Kindlin-2 was confirmed by qPCR. Four RNA samples (Kindlin-2 SiRNA transfected TM4 and 15P-1 cell samples, control SiRNA transfected TM4 and 15P-1 cell samples) were sequenced on the HiSeq.2000 sequencing platform (Illumina). Differential gene expression was performed with Novomagic webtool (https://magic.novogene.com/). Genes with a false discovery rate (FDR) < 0.05 were considered to be differentially expressed. Pathway analysis (KEGG, Kyoto Encyclopedia of Genes and Genomes) was performed with Annotation, Visualization and Integrated Discovery (DAVID) (https://david.ncifcrf.gov/) using down regulated genes (*P* < 0.05). Gene Ontology (GO) enrichment analysis was performed with GENE ontology unifying biology (http://geneontology.org/docs/go-enrichment-analysis/) using all related genes (*P* < 0.05).

#### Quantitative PCR

Total RNAs were isolated using the Trizol reagent (Invitrogen) following the manufacturer’s protocol. 1 μg of RNA was subjected to reverse transcription using a PrimeScript RT reagent Kit (Takara, China). Quantitative real-time PCR (qRT-PCR) was performed in triplicate using SYBR Green PCR Master Mix and a Light-Cycler 480 (Roche Diagnostics). A 10 μl reaction mixture was used for qRT-PCR containing 10 ng cDNA, 0.5 mM of each of the primers and 5 μl 2X Green Premix Ex Taq (TaKaRa, China). After the cycling protocol, a melting curve was generated as specificity control for primers. The PCR efficiency of each individual samples was 85%-115%. Gene expression was normalized to GAPDH and calculated using the 2^-ΔΔCt^ method. The primers used in the present study are listed as follows:

*Kindlin-2* forward: acctgctcatcagctgaacc; *Kindlin-2* reverse: gcgcgtactgcttctcgtta;

*GAPDH* forward: aggtcggtgtgaacggatttg; *GAPDH* reverse: tgtagaccatgtagttgaggtca;

*Jam2* forward:gcgcccgcgtagatgg; *Jam2* reverse: ccagtctggaggaggtagtctt;

*Dlg2* forward: ctgacagagggaacggatgc; *Dlg2* reverse: gcacaagttctggccctctt;

*Bmp4* forward: gcaacccagcctgagtatct; *Bmp4* reverse: atggcactacggaatggctc;

*Wnt4* forward: ccgggcactcatgaatcttc; *Wnt4* reverse: cacccgcatgtgtgtcaag;

*Wnt10b* forward: gacgccggggaagcg; *Wnt10b* reverse: ggtgggcgagtcagtcag;

*TGFβ2* forward: tcgaccatggatcagtttatgcg; *TGFB2* reverse: ccctggtactgttgtagatgga;

*Cypr61* forward: tctgtgaagtgcgtccttgt; *Cypr61* reverse: cgcagtatttgggccggtat.

#### Quantification of cells

For quantitation of Sox9-positive Sertoli cells, GCNA-positive germ-cells, six random tubules were counted (*n* = 3 mice per genotype). To determine the proliferated and apoptotic cells, the number of Ki67-positive cells and Tunel-positive cells (apoptotic cells) per tubules were measured (*n* = 6 tubules per section, *n* = 3 mice per genotype). All cells were counted under images.

#### Co-immunoprepiation (Co-IP)

The cells were lysed in RIPA buffer containing protease inhibitor cocktail and precleared with protein-A/G agarose (Sigma Aldrich). For each immunoprecipitation assay, 2 mg protein was used, and 2 µg of indicated antibodies was added for each reaction. The immune complex was incubated overnight on an orbital shaker at 4 °C and then incubated with 50 µl of 50% protein-A/G agarose. The beads were then washed with RIPA buffer three times, resolved by SDS-PAGE and analyzed by western blot.

#### Statistical analysis

All values and error bars in graphs are means ± standard error of the mean (Means ± SD). Comparisons between two groups were made using two-tailed Student’s *t* test. Statistical significance was considered for *p < 0.05, **p < 0.01 and ***p < 0.001. Analyses were carried out using GraphPad Prism 6.0 (GraphPad Software Inc., La Jolla, CA).

## Supplementary information

Cell Death X Disease-0413-Chi-etal-Supplementary Figure legends

Supplementary Figure 1.

Supplementary Figure 2.

Supplementary Figure 3.

## References

[1] Zhan J (2014). Kindlin-2 expression in adult tissues correlates with their embryonic origins. Sci. China Life Sci..

[2] Montanez E (2008). Kindlin-2 controls bidirectional signaling of integrins. Genes Dev..

[3] Zhan HX, Xu JW, Wu D, Zhang TP, Hu SY (2015). Pancreatic cancer stem cells: new insight into a stubborn disease. Cancer Lett..

[4] Qi L (2015). Kindlin-2 interacts with alpha-actinin-2 and beta1 integrin to maintain the integrity of the Z-disc in cardiac muscles. FEBS Lett..

[5] He X (2020). Kindlin-2 deficiency induces fatal intestinal obstruction in mice. Theranostics.

[6] Schlegel PN (1997). Testicular sperm extraction with intracytoplasmic sperm injection for nonobstructive azoospermia. Urology.

[7] Ramphul, K. & Mejias, S. G. Sertoli-cell-only syndrome. (StatPearls, Treasure Island, FL, 2018).

[8] Sharpe RM, McKinnell C, Kivlin C, Fisher JS (2003). Proliferation and functional maturation of Sertoli cells, and their relevance to disorders of testis function in adulthood. Reproduction.

[9] Chojnacka K, Zarzycka M, Mruk DD (2016). Biology of the sertoli cell in the fetal, pubertal, and adult mammalian testis. Results Probl. Cell Differ..

[10] Franca LR, Hess RA, Dufour JM, Hofmann MC, Griswold MD (2016). The Sertoli cell: one hundred fifty years of beauty and plasticity. Andrology.

[11] Mruk DD, Cheng CY (2015). The mammalian blood-testis barrier: its biology and regulation. Endocr. Rev..

[12] Xia W, Wong CH, Lee NP, Lee WM, Cheng CY (2005). Disruption of Sertoli-germ cell adhesion function in the seminiferous epithelium of the rat testis can be limited to adherens junctions without affecting the blood-testis barrier integrity: an in vivo study using an androgen suppression model. J. Cell Physiol..

[13] Smith BE, Braun RE (2012). Germ cell migration across Sertoli cell tight junctions. Science.

[14] Munell F, Suarez-Quian CA, Selva DM, Tirado OM, Reventos J (2002). Androgen-binding protein and reproduction: where do we stand?. J. Androl..

[15] Gill-Sharma MK (2018). Testosterone retention mechanism in Sertoli cells: a biochemical perspective. Open Biochem. J..

[16] Holdcraft RW, Braun RE (2004). Androgen receptor function is required in Sertoli cells for the terminal differentiation of haploid spermatids. Development.

[17] Wolski KM, Haller E, Cameron DF (2005). Cortactin and phagocytosis in isolated Sertoli cells. J. Negat. Results Biomed..

[18] Bottcher RT (2017). Kindlin-2 recruits paxillin and Arp2/3 to promote membrane protrusions during initial cell spreading. J. Cell Biol..

[19] Li B (2019). Integrin-interacting protein Kindlin-2 induces mammary tumors in transgenic mice. Sci. China Life Sci..

[20] Fu V, Plouffe SW, Guan KL (2017). The Hippo pathway in organ development, homeostasis, and regeneration. Curr. Opin. Cell Biol..

[21] Yu FX, Zhao B, Guan KL (2015). Hippo pathway in organ size control, tissue homeostasis, and cancer. Cell.

[22] Hao Y, Chun A, Cheung K, Rashidi B, Yang X (2008). Tumor suppressor LATS1 is a negative regulator of oncogene YAP. J. Biol. Chem..

[23] Oka T, Mazack V (2008). Sudol M. Mst2 and Lats kinases regulate apoptotic function of Yes kinase-associated protein (YAP). J. Biol. Chem..

[24] Bossuyt W (2014). An evolutionary shift in the regulation of the Hippo pathway between mice and flies. Oncogene.

[25] An Z (2010). Kindlin-2 is expressed in malignant mesothelioma and is required for tumor cell adhesion and migration. Int. J. Cancer.

[26] Gong X (2010). Kindlin-2 controls sensitivity of prostate cancer cells to cisplatin-induced cell death. Cancer Lett..

[27] Levasseur A, Paquet M, Boerboom D, Boyer A (2017). Yes-associated protein and WW-containing transcription regulator 1 regulate the expression of sex-determining genes in Sertoli cells, but their inactivation does not cause sex reversal. Biol. Reprod..

[28] Song J (2019). Kindlin-2 Inhibits the Hippo signaling pathway by promoting degradation of MOB1. Cell Rep..

[29] Guo L (2018). Kindlin-2 regulates mesenchymal stem cell differentiation through control of YAP1/TAZ. J. Cell Biol..

[30] Boggiano JC, Fehon RG (2012). Growth control by committee: intercellular junctions, cell polarity, and the cytoskeleton regulate Hippo signaling. Dev. Cell.

