# Supplementary material for: Kindlin-2 in Sertoli cells is essential for testis development and male fertility in mice
Source: Cell Death Dis. 2021 Jun 11;12(6):604. doi: 10.1038/s41419-021-03885-4 (PMC8196014; doi:10.1038/s41419-021-03885-4)
Supplement: Supplementary file 1 — Cell Death X Disease-0413-Chi-etal-Supplementary Figure legends [file 41419_2021_3885_MOESM1_ESM.doc]

**Supplementary Figure Legends**

**Title: Kindlin-2 in Sertoli cells is essential for testis development and male fertility in mice**

**Supplementary Figure 1: Generation of Kindlin-2 Sertoli cell-specific knockout mice.**

**(A)** Schematic representation of targeting vector and the targeting strategy for generating a Kindlin-2 null allele. Structures of the Kindlin-2 floxed alleles (*Kindlin-2^f/f^*) and the schematic representation of generation of recombined alleles by crossing with Amh-Cre transgenic mice. The exons are numbered and the position of primers for genotyping are shown. The meaning of icons is shown on the low right panel as an inset.

**(B)** PCR genotyping of *Kindlin-2^f/f^*, *Amh-Cre*; *Kindlin-2^f/+^*, *Amh-Cre*; *Kindlin-2^f/f^* and WT flox allele using specific primers.

**(C)** Schematic representation of *ROSA26^mTmG^* targeted allele and the targeting strategy for generating *Amh-Cre*; *ROSA26^mTmG^* mice.

**(D)** Confirm the specificity and efficiency of Cre expression in Amh-Cre mice. *ROSA26^mTmG^*; *Kindlin-2^f/f^* mice were crossed with *Amh-Cre* and *Amh-Cre*; *Kindlin-2^f/+^* mice to generate *Amh-Cre*; *ROSA26^mTmG^*; *Kindlin-2^f/+^* and *Amh-Cre*; *ROSA26^mTmG^*; *Kindlin-2^f/f^* mice, respectively. *ROSA26^mTmG^*; *Kindlin-2^f/f^* mice were used as a negative control. Mice were sacrificed at 8W. Cryosections were stained with 1 mg/ml DAPI. Scale bar, 50 μm.

**(E)** Body size and external genitalia gross morphology of *Amh-Cre*; *Kindlin-2^f/f^* (KO) and littermates with different genotypes at 8W.

**(F, G)** Gross morphology of testis (F) and epididymis (G) from Kindlin-2 KO and littermates with different genotypes at 8 w. Scale bar, 5 mm

**Supplementary Figure 2:**

**Sertoli cell specific knockout of Kindlin-2 in mice does not induce destruction of seminiferous tubules and testicular dysplasia at day 2.**

1. HE staining of 2 day WT and KO mice testis. The seminiferous tubules were grossly normal in KO mice compared with WT mice. Scale bar, 50 μm.
2. Hematoxylin and eosin-staining (HE) and immunohistochemistry (IHC) of WT1 (Sertoli cell nucleus marker) in 4 w testes (magnification of Figure 2C). ① Spermatogonia, ② Primary spermatocytes, ③ Secondary spermatocytes, ④ Spermatids, ⑤ Spermatozoa, ⑥ Sertoli cells, ⑦ Stromal cells.

**Supplementary Figure 3: RNA sequencing analyses demonstrate that depletion of Kindlin-2 in Sertoli cells affects cell junction, adhesion and organ development.**

**(A, B)** The Molecular Function, Cellular Component and Biological Process GO enrichment terms of regulated genes (A) and down-regulated genes (B) by using DAVID online tool. GO0098636, protein complex involved in cell adhesion; GO0098634, protein binding involved in cell-matrix adhesion; GO0007300, transcription factor activity. Enrichment scores are shown as -log10 (P-value).

**(C)** A heat map analysis of cell-cell junction (GO0005911) related genes. Blue and red represent low and high expression levels respectively.
