# Supplementary figures and images for: Kindlin-2 in Sertoli cells is essential for testis development and male fertility in mice
Source: Cell Death Dis. 2021 Jun 11;12(6):604. doi: 10.1038/s41419-021-03885-4 (PMC8196014; doi:10.1038/s41419-021-03885-4)

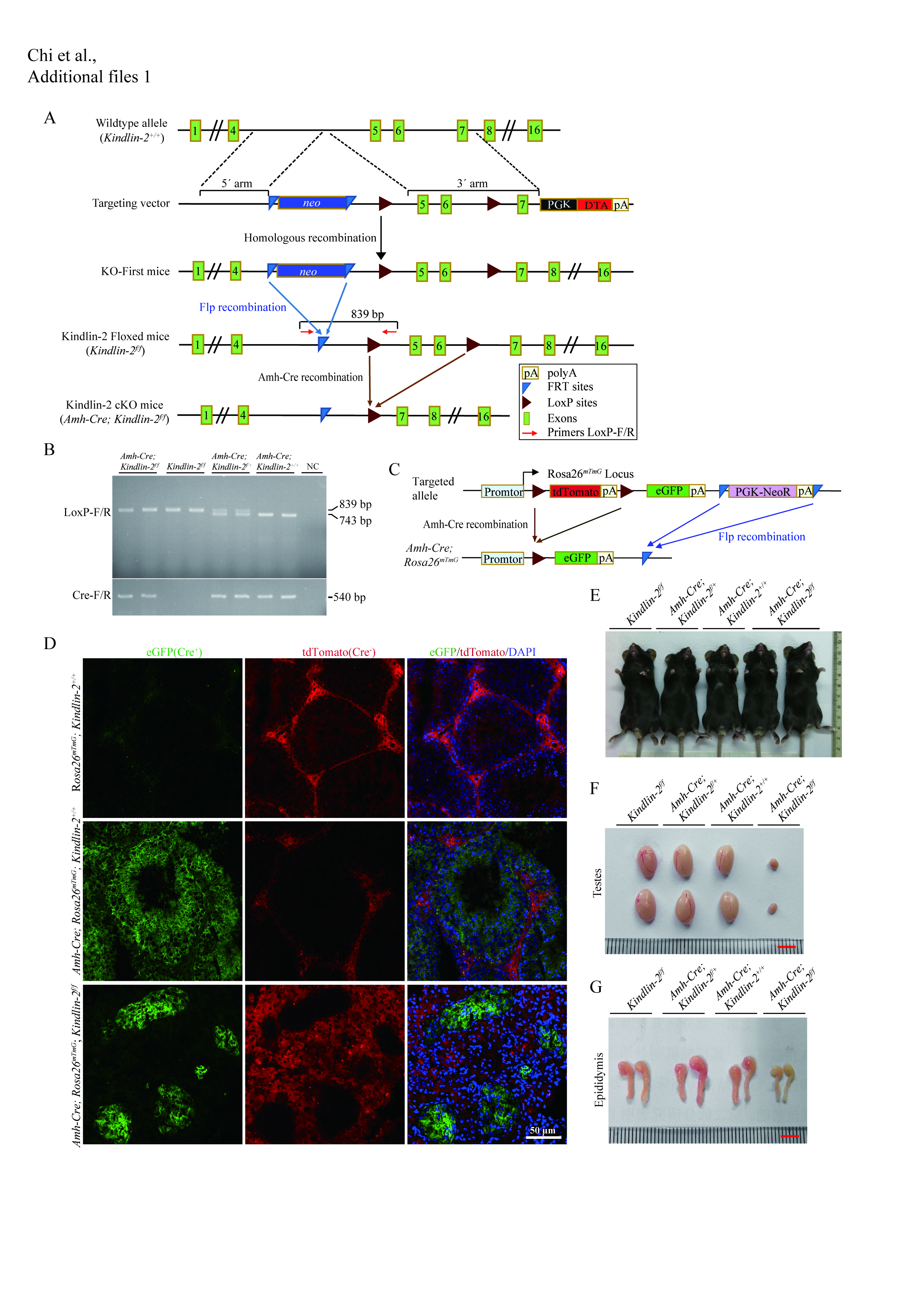

Supplement: Supplementary file 2 — Supplementary Figure 1. [file 41419_2021_3885_MOESM2_ESM.tif]

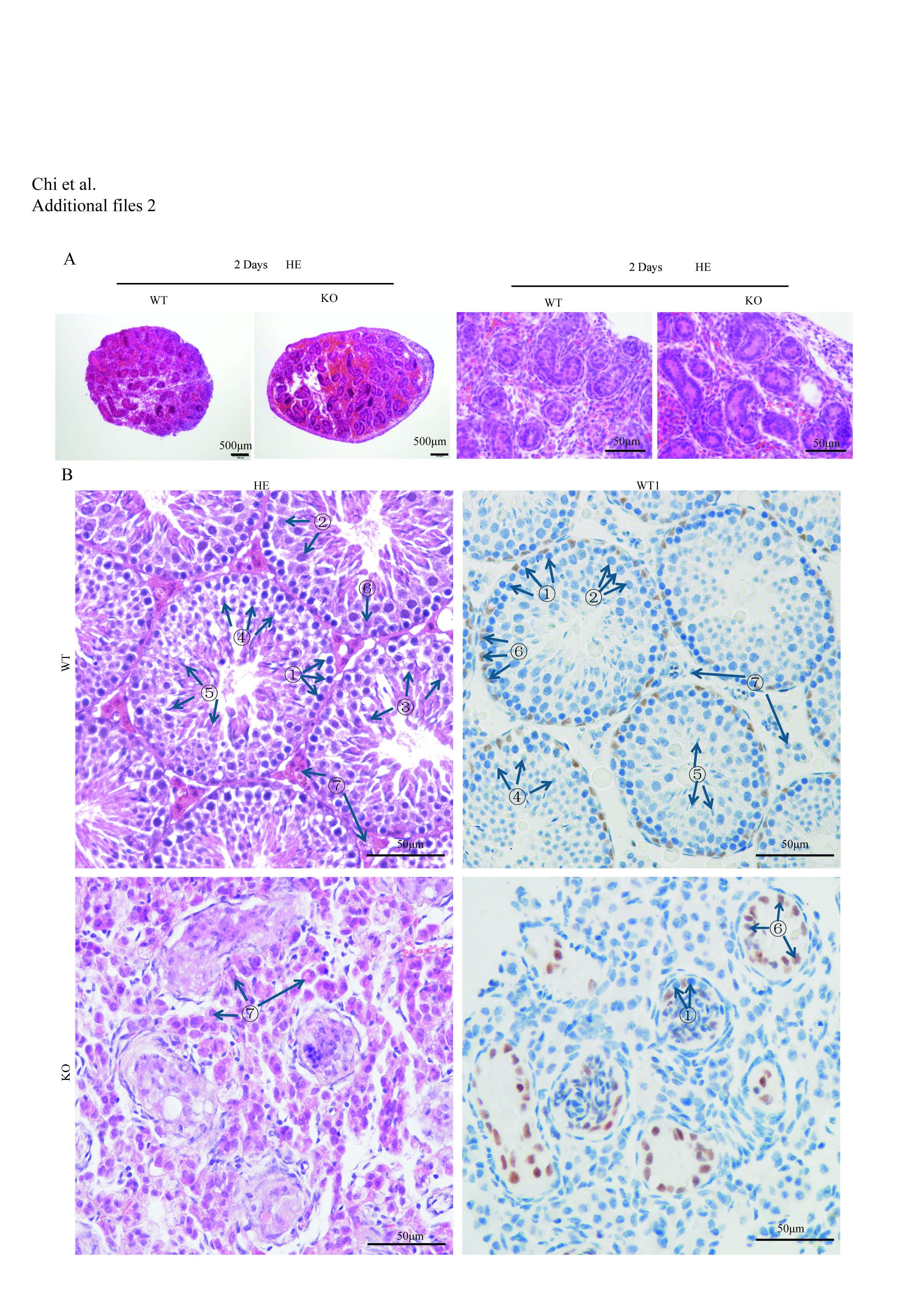

Supplement: Supplementary file 3 — Supplementary Figure 2. [file 41419_2021_3885_MOESM3_ESM.tif]

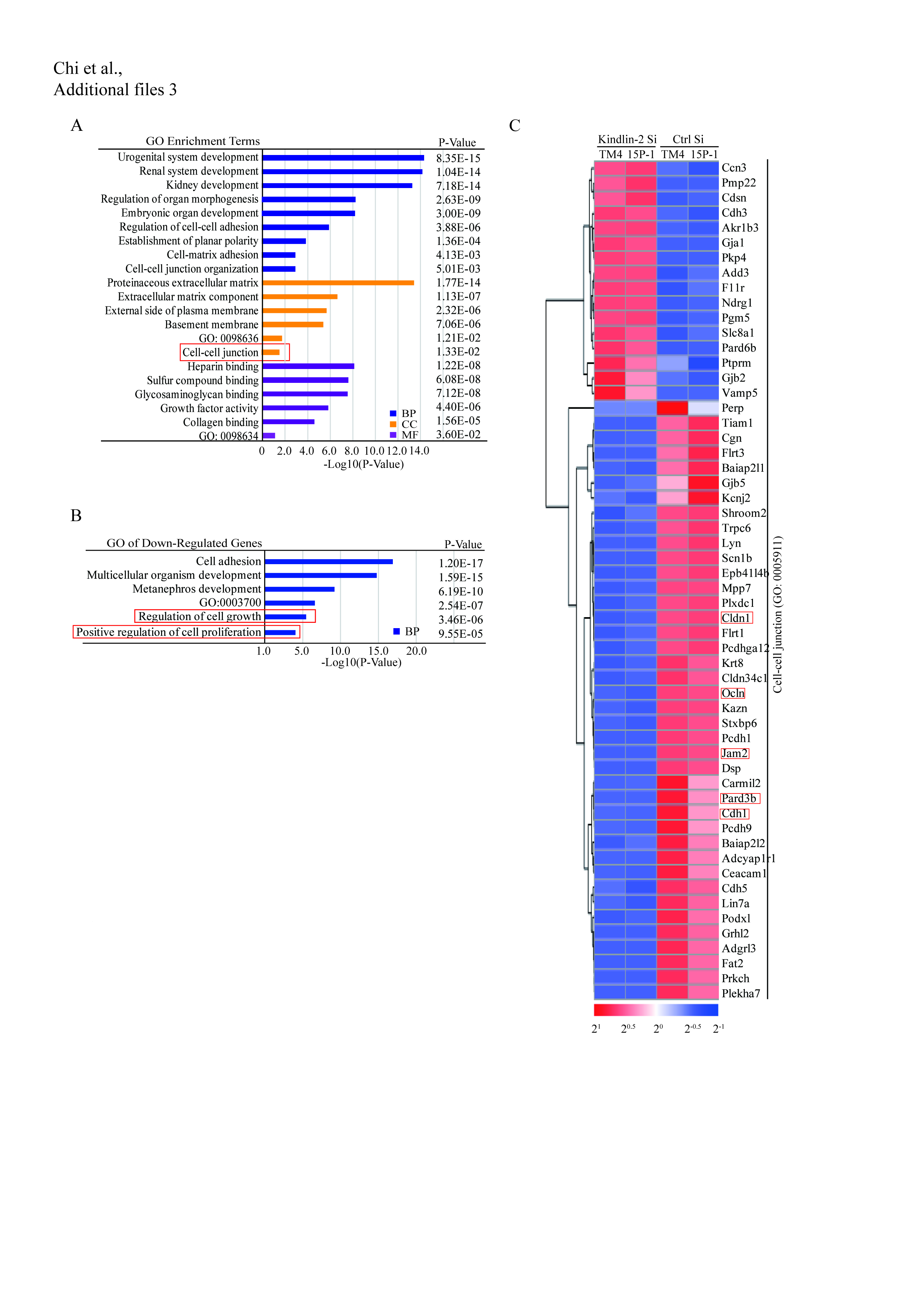

Supplement: Supplementary file 4 — Supplementary Figure 3. [file 41419_2021_3885_MOESM4_ESM.tif]
